# *Aedes* Mosquitoes and *Aedes*-Borne Arboviruses in Africa: Current and Future Threats

**DOI:** 10.3390/ijerph15020220

**Published:** 2018-01-28

**Authors:** David Weetman, Basile Kamgang, Athanase Badolo, Catherine L. Moyes, Freya M. Shearer, Mamadou Coulibaly, João Pinto, Louis Lambrechts, Philip J. McCall

**Affiliations:** 1 Department of Vector Biology, Liverpool School of Tropical Medicine, Pembroke Place, Liverpool L3 5QA, UK; david.weetman@lstmed.ac.uk; 2 Centre for Research in Infectious Diseases, Yaoundé PO Box 13501, Cameroon; kamgang_d@yahoo.fr; 3 Laboratoire d’Entomologie Fondamentale et Appliquée (LEFA), Université Ouaga 1 Pr Joseph Ki-Zerbo, Ouagadougou 03 BP 7021, Burkina Faso; a.badolo@gmail.com; 4 Big Data Institute, Li Ka Shing Centre for Health Information and Discovery, University of Oxford, Oxford OX3 7LF, UK; clmoyes@well.ox.ac.uk (C.L.M.); freya.m.shearer@gmail.com (F.M.S.); 5 University of Sciences, Techniques and Technologies of Bamako, Bamako BP 1805, Mali; doudou@icermali.org; 6 Global Health and Tropical Medicine (GHTM), Instituto de Higiene e Medicina Tropical (IHMT), Universidade Nova de Lisboa (UNL), Rua da Junqueira 100, 1349-008 Lisbon, Portugal; JPinto@ihmt.unl.pt; 7 Insect-Virus Interactions, Department of Genomes and Genetics, Institut Pasteur, 75015 Paris, France; louis.lambrechts@pasteur.fr; 8 Centre National de la Recherche Scientifique, Unité Mixte de Recherche 2000, 75015 Paris, France

**Keywords:** *Aedes aegypti*, *Aedes albopictus*, *Aedes formosus*, Zika, dengue, chikungunya, yellow fever, vector

## Abstract

The Zika crisis drew attention to the long-overlooked problem of arboviruses transmitted by *Aedes* mosquitoes in Africa. Yellow fever, dengue, chikungunya and Zika are poorly controlled in Africa and often go unrecognized. However, to combat these diseases, both in Africa and worldwide, it is crucial that this situation changes. Here, we review available data on the distribution of each disease in Africa, their *Aedes* vectors, transmission potential, and challenges and opportunities for *Aedes* control. Data on disease and vector ranges are sparse, and consequently maps of risk are uncertain. Issues such as genetic and ecological diversity, and opportunities for integration with malaria control, are primarily African; others such as ever-increasing urbanization, insecticide resistance and lack of evidence for most control-interventions reflect problems throughout the tropics. We identify key knowledge gaps and future research areas, and in particular, highlight the need to improve knowledge of the distributions of disease and major vectors, insecticide resistance, and to develop specific plans and capacity for arboviral disease surveillance, prevention and outbreak responses.

## 1. Introduction and Overview

The crisis following the Zika epidemic across South America in 2015–2016 and the newly recognised severe neurological consequences of infection have raised global awareness of *Aedes*-borne arboviral diseases. Zika has also belatedly turned the scientific community’s attention to Africa, where this virus was originally identified [1] and where the other *Aedes*-transmitted arboviruses also occur. Despite its global importance [2], the extent of the dengue burden in Africa remains unknown [3], as does the true burden of yellow fever, though tens of thousands die annually in Africa and the threat of outbreaks remains high [4,5], even with availability of an effective vaccine [6]. Moreover, the risk of exporting yellow fever from Africa remains a concern, particularly to immunologically naïve Asian populations [7,8].

Why was so little attention paid to *Aedes*-borne arboviruses in Africa, until they led to outbreaks in locations outside of Africa? One explanation is that these viruses constitute greater public health threats for immunologically-naïve non-African human populations. A second is that the magnitude of the burden of malaria and many neglected tropical diseases in Africa detract attention from *Aedes*-borne infections. A third and related factor is that accurate identification of arboviral infections in resource-poor settings is challenging, and in Africa, many fevers of unknown origin tend to be recorded as malaria [7]. Hence, the major arboviral infections remain neglected in Africa, while receiving significant attention and resources for control elsewhere.

In common with Zika [8], chikungunya [9], yellow fever [10], and perhaps also dengue [11], *Aedes aegypti* almost certainly originated in Africa [12,13,14]. Globally *Ae. aegypti* is the primary vector of all of these viruses, but a range of African *Aedes* species are competent and epidemiologically-significant vectors. An important addition to this vector fauna is *Aedes albopictus*, introduced into Africa less than 30 years ago and still increasing its range [15]. 

Adaptation of arboviruses to novel vectors can occur rapidly and can have significant consequences. A single amino acid substitution facilitated efficient transmission of the chikungunya virus by *Ae. albopictus* [16], permitting establishment of local transmission in many new regions beyond Africa, following the 2005–2006 outbreak on the island of La Réunion. Simple genetic changes can also affect the virus’s interaction with the vertebrate host, with uncertain but potentially severe consequences [17]. The Zika virus also can be transmitted through sexual intercourse, though the epidemiological importance of this mode of transmission remains to be determined [18].

Control of Zika and other *Aedes*-transmitted arboviruses is a major 21st century challenge for global public health, exacerbated by widespread insecticide resistance in the vectors [19] and a relentless growth in urban environments [20]. This is especially true in the resource-poor sub-Saharan nations, which have the lowest collective Gross Domestic Product (GDP) of any region [21]. A thorough exploration of the burden, transmission biology and control of *Aedes*-transmitted arboviruses is essential to define, understand and prepare for potential future threats in Africa and worldwide [22,23]. Here, we review current information on these key topics for *Aedes* vectors and the major arboviruses they transmit in Africa, highlighting knowledge gaps, and recommending priorities for further investigation.

## 2. The Burden of *Aedes*-Borne Viral Diseases in Africa

**Figure 1:**
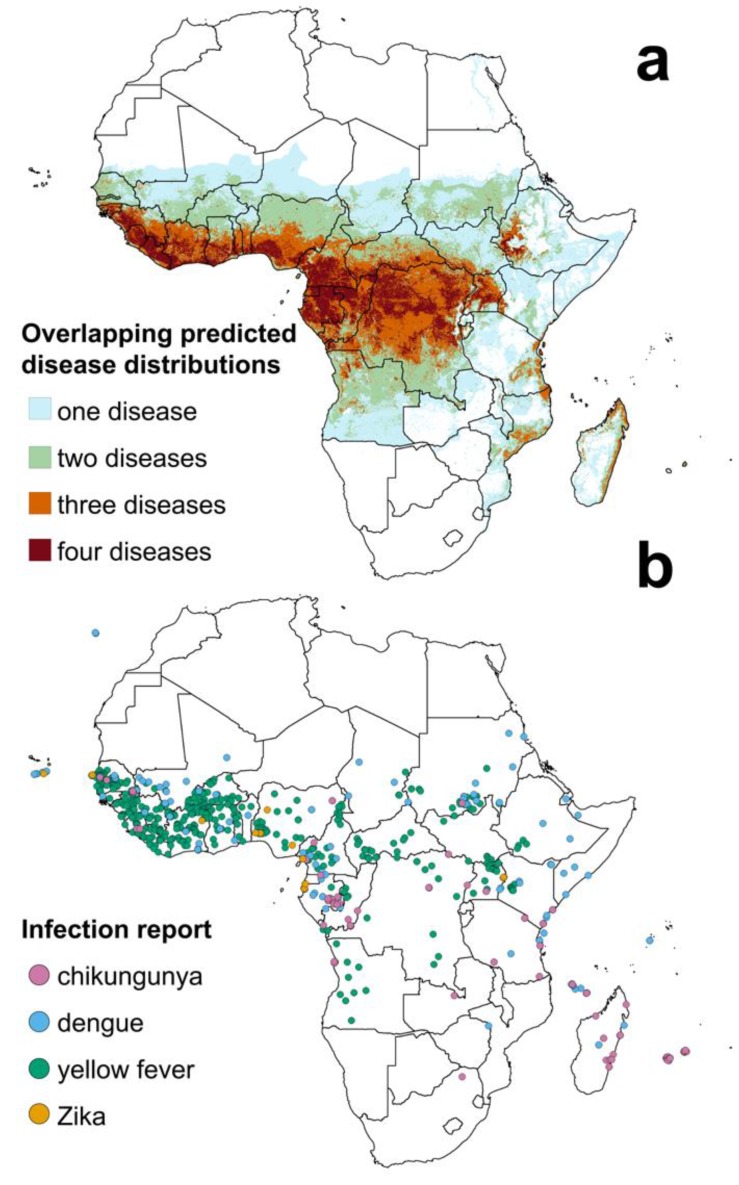
The distributions of chikungunya, dengue, yellow fever and Zika virus infections in humans in Africa. (**a**) Areas at risk of one, two, three or all four infections; map generated as described in Supplementary Methods. (**b**) Locations of reported infections (symptomatic and non-symptomatic) of dengue, chikungunya, Zika and yellow fever [24,25,26,27,28].

The extent of chikungunya, dengue and Zika virus distributions in Africa is largely unknown, and our knowledge of the variation in transmission risk within these zones, as well as within the yellow fever risk zone [6], is hampered by a lack of disease data. Ecological models have been used to estimate the spatial distributions of each virus [2,24,25,26], and we have used these maps to generate comparable estimates for the population at risk of infection (PAR) (Supplementary Materials). Table 1 shows the estimated African PAR for chikungunya, dengue, yellow fever and Zika [24,25,26,27,28]. The value for yellow fever was adjusted to take account of vaccination campaigns conducted in parts of Africa and shows the much lower PAR for this disease since vaccination began [29]. Individual disease distributions overlap (Figure 1), and an estimated 831 million people were living in an area at risk of at least one of these arbovirus infections in 2015 (Table 1). However, these estimates are based on predicted distributions within unknown disease ranges: actual PAR values cannot be estimated with certainty until the extent of each disease has been defined and more case data are collected across the continent. Furthermore, the number of people living in an area of risk who are actually protected by life-long immunity conferred by previous infection with the chikungunya virus, Zika virus and each serotype of the dengue virus is unknown but could change the PAR figures dramatically [30,31,32]. Outbreaks of all four diseases are characterised by large spikes in the number of cases, often preceded and followed by periods when no new cases are reported [33]. Therefore, the values shown (Table 1 and Figure 1) represent the presence of a transmission risk at some point in time over a number of years, rather than a year-round or seasonal risk. As the uncertainty in the PAR highlights, there is an urgent need to improve data availability and quality in order to map more accurately the disease burden in Africa.

## 3. Range and Distribution of *Aedes* Vector Species in Africa

**Figure 2:**
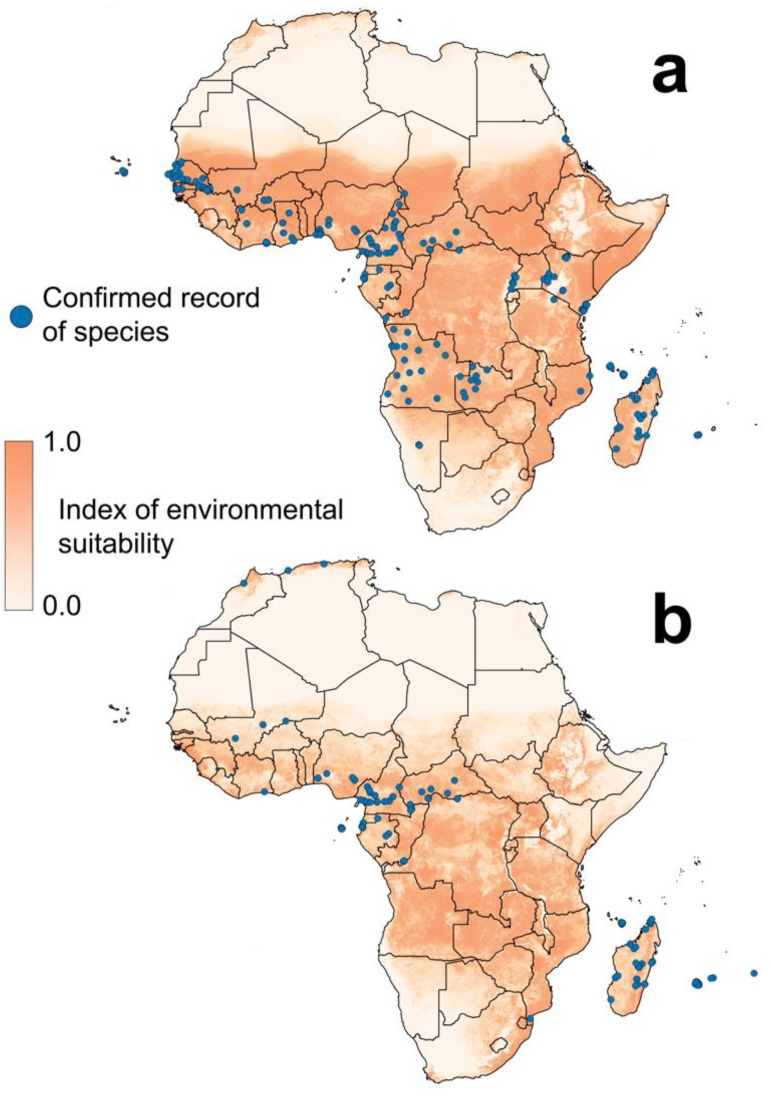
Reports of mosquito occurrence and areas of predicted environmental suitability for *Aedes albopictus* and *Ae. aegypti* in Africa [36,37,38,39,40,41,42,43,44,45,46,47,48,49,50,51,52,53,54,55,56,57,58,59,60,61,62,63,64]: (**a**) *Ae. aegypti*; *(***b**) *Ae. albopictus.*

African ranges are unknown, even for the two major *Aedes* vector species, and there are insufficient records to allow their definition either by expert opinion or any semi-quantitative method. Suitable environments for *Ae. albopictus* and *Ae*. *aegypti* within Africa have been predicted using ecological models (Figure 2) [34], and though the maps indicate extensive areas of suitability and a large potential for sympatric occurrence, these estimated distributions need to be treated with caution. Maps show locations where the species could potentially occur but not necessarily where they have been found. For example, much of sub-Saharan Africa is predicted to be suitable for *Ae*. *albopictus*, but records remain patchy [35,36,37,38,39,40,41,42,43,44,45,46,47,48,49,50,51,52,53,54,55,56,57,58,59,60,61,62,63,64]. Since the worldwide records of each species were compiled, new studies in Africa have reported *Ae. aegypti* in Ghana [51], Mozambique [55] and Namibia [59], and *Ae. albopictus* in Mali [57], Morocco [39], Mozambique [50] and São Tomé and Príncipe [62]. 

The areas of predicted suitability shown in Figure 2 are based on relationships with environmental and socio-economic variables. Studies using global records of each species found that temperature is the most influential predictor and that precipitation, vegetation indices and urban land cover also play a role [33,65]. Few studies have looked at these relationships in African populations, but temperature is still likely to be important [66,67] and rainfall is strongly linked to *Aedes* vector abundance in Kenyan populations [68]. Specific landscape factors are more likely to vary between different regions of the world and the influence of some of these have been studied in African locations. In Central Africa, *Ae. albopictus* preferred man-made breeding sites (tyres and tanks) surrounded by vegetation whereas *Ae. aegypti* preferred man-made breeding sites in neighbourhoods with higher building density [69,70]. Similarly, in urban Cameroon, *Ae. aegypti* was more abundant in downtown environments in the dry season whereas *Ae. albopictus* was more prevalent in the suburbs in all seasons [71]. Studies in rural areas have found that within oil palm-dominated landscapes in Côte d’Ivoire *Ae. aegypti* was more abundant in polycultures than monocultures [72].

Large gaps in reporting of each species can be seen in Figure 2, but vector presence can be inferred from reports of locally acquired arbovirus infections (Figure 1b). These case reports show infections occurring in a number of countries where no vector data are available, implying that one of more of the arbovirus vectors is present. Which vectors might be found in these locations can only be addressed by field surveys, and priority should be given to the countries reporting arbovirus infections but no recent reports or records of vector species. Currently these are Guinea, Guinea Bissau, Sierra Leone, Liberia, Togo, Chad, South Sudan, Ethiopia, Eritrea, Djibouti, Somalia. 

## 4. Vectorial Capacity of Different *Aedes* Species in Africa

### 4.1. Aedes aegypti vs. Aedes albopictus

*Aedes aegypti* is considered the main vector of dengue viruses worldwide, largely attributable to its higher vector competence and stronger host preference for humans compared to *Ae. albopictus* [73]. However, *Ae. albopictus* has been a driving force in the worldwide emergence of chikungunya virus since 2004 [74], and in Central Africa it is considered to have played a key role in the 2007 emergence of dengue, chikungunya [75,76] and possibly Zika [77]. Moreover, its ongoing range expansion across Africa has the potential to increase the arbovirus transmission risk to areas far from urbanisation [78]. Improved knowledge of *Ae. albopictus* distribution and vectorial capacity in Africa is required to enhance arbovirus surveillance and prevention [79].

### 4.2. Sylvatic vs. Domestic Forms of Aedes aegypti

*Aedes aegypti* is believed to have originated in Africa from a generalist, zoophilic tree-hole breeder [12]. Outside Africa, *Ae. aegypti* populations are exclusively found in close association with humans in the domestic environment, and this domestic form of *Ae. aegypti* may have originated from a population that adapted to (i) breeding in artificial water storage containers and (ii) biting humans [80]. Domestication events involving human-feeding specialization may have occurred both outside Africa as part of the species’ expansion, and within Africa [81], possibly, but perhaps not exclusively, on the West African coast [82].

African *Ae. aegypti* breed in both the domestic environment and in the ancestral sylvatic habitat. Whereas domestic *Ae. aegypti* larvae develop in artificial containers (e.g., tyres, discarded containers, jars, flower pots, metal drums) within or in close proximity to human habitation, larvae of the sylvatic ecotype are found in natural breeding sites (e.g., rock pools, tree holes, plant axils, fruit husks) in forested areas [80]. Interestingly, larvae of the two *Ae. aegypti* ecotypes are exposed to different bacterial communities in their respective breeding sites, potentially resulting in differences in vectorial capacity [83]. Classically, two morphological subspecies were described in Africa that broadly correspond with these ecotypes: *Ae. aegypti aegypti* and *Ae. aegypti formosus*. However, there is evidence that *Ae. aegypti formosus* is increasingly found in urban environments [84], and the diagnostic morphological characters (presence/absence of white abdominal scaling patterns [85]) often form a continuum [86]. Similarly, clear genetic boundaries are absent, presumably as a result of extensive current or recent historical gene flow [87,88]. The picture may be complicated further by introductions of domestic *Ae. aegypti* into Africa and interbreeding with local populations [89,90]. The result is a lack of clear correlation between morphology, genetics and ecology [81,90,91], which calls into question the utility of the simple division of the diverse African *Ae. aegypti* fauna into the *aegypti* and *formosus* subspecies [80,84].

African *Ae. aegypti* have traditionally been considered less competent for flaviviruses than *Ae. aegypti* from the rest of the world [92,93]. A global survey of 28 populations found that sylvatic *Ae. aegypti* populations from West Africa were the least susceptible to yellow fever virus infection [94]. However, more recent observations indicated that vector competence of African *Ae. aegypti* is extremely variable and depends on specific pairings of mosquito population and viral isolate [76,95,96,97,98]. Such genotype-by-genotype specificity has been widely documented in other host-pathogen systems [99]. 

### 4.3. Other Aedes Species and Potential for Emergent Transmission Cycles

Additional *Aedes* species play a critical role in arbovirus transmission cycles in Africa because (i) they are involved in sylvatic arbovirus transmission cycles and/or (ii) they bridge sylvatic and human transmission cycles. For example, *Aedes africanus* is considered the main sylvatic vector of yellow fever virus in Africa [100] and can also act as a bridge vector to humans, together with *Aedes bromeliae*, *Aedes furcifer*, *Aedes taylori*, *Aedes luteocephalus*, *Aedes metallicus*, *Aedes opok*, *Aedes vittatus*, and species of the *Aedes simpsoni* complex [101]. Sylvatic dengue viruses in Africa are transmitted among non-human primates by *Ae. furcifer* and *Ae. luteocephalus*, and usually cross over to humans through biting by *Ae. furcifer* [101]. Bridge vectors may initiate a human outbreak, but large epidemics typically occur only when virus transmission involves urban populations of *Ae. aegypti* or *Ae. albopictus*, though there may have been exceptions: Haddow [102] described *Ae. simpsoni* (=*Ae. bromeliae* [15]) as being “the principal vector in the greatest known African yellow fever epidemic”, which occurred in Ethiopia in the early 1960s. 

The majority of these *Aedes* vector species are found in rural or forest areas, and so are less likely to present a threat in the urban environments where *Ae. aegypti* populations thrive. Nonetheless, increasing erosion of their natural habitats could lead to greater contact with humans, and/or formerly obligate sylvatic species might adapt to new urban environments and hosts, potentially with a greater role as vectors [103]. Many readily feed on both domestic and wild animals including primates, as well as humans, hence their potential importance as bridging vectors [96,104,105,106]. Although a sylvatic dengue transmission cycle is known in restricted locations like eastern Senegal [107,108], it is unclear how important it is across the continent of Africa. All of the focal arboviral diseases circulate among non-human primates in Africa, though the true role of animal reservoirs in the epidemiology of human disease remains to be determined [109]. 

Less is known about the African vectors of Zika, and although multiple *Aedes* species from across Africa have been found naturally infected with the Zika virus, this is also true for non-*Aedes* species, and cannot be translated into vector competence [110,111]. In a rare demonstration, a small proportion of *Ae. vittatus* and *Ae. luteocephalus* were found to harbour the virus in their salivary glands following artificial infection with Zika strains, suggesting some potential for transmission [110]. With a growing body of evidence from elsewhere showing the Zika virus’ capacity for development and transmission by species in addition to *Ae. aegypti* and *Ae. albopictus* [112], further studies to test competence in the African vectors from which it has been isolated should be prioritised.

Better knowledge of African transmission cycles is important to determine the risk of viruses like Zika or chikungunya establishing similar sylvatic cycles in geographic regions where they have newly arrived [113,114]. Recent reports from Kenya suggest that human infections may be a source of ‘spillback’ infections in baboon populations [85], demonstrating the range of potential consequences following introduction of novel viruses or strains. Clearly, assessing the risk of human infection by arboviruses will require more in-depth studies on the biology of sylvatic *Aedes* spp. in Africa [104,107].

## 5. Vector Surveillance and Control of *Aedes* species in Africa

The majority of *Aedes* spp. control studies have focused on dengue outbreaks in urban sites in Asia or Latin America, where transmission by *Ae. aegypti* and/or *Ae. albopictus* is exclusively human to human [115,116]. Indeed, a recent systematic review of the evidence for effectiveness of vector control in reducing dengue virus transmission included 41 trials, but none were from Africa [117].

The widespread use of the insecticide DDT (dichlorodiphenyltrichloroethane) in *Ae. aegypti* eradication programmes, that led to its near elimination from South America by the late 1960s, was never replicated in Africa [4,118]. Subsequently, factors such as increased international freight and travel, urbanization, and vector control strategies and tools poorly suited to urban environments or hampered by insecticide resistance [119] have resulted in the global explosion of dengue. Yellow fever outbreaks can be prevented or controlled by mass vaccine distribution [120], but no vaccines are available for chikungunya and Zika, and the licensed dengue vaccine offers incomplete and serotype-specific protection [121]. Consequently, outbreak prevention and response remain reliant on vector control.

Preventing outbreaks, or mitigating their impact, is challenging even for those countries that have made considerable investment in capacity after fighting against dengue outbreaks for decades [122]. This scenario and the associated challenges are exacerbated in African countries where recognition of dengue outbreaks has started relatively recently; dengue is often not a reported infection, even if correctly diagnosed, and where surveillance and response strategies may be absent or improperly developed [123,124,125,126]. Rolling out effective surveillance plans for *Aedes-*borne diseases, particularly dengue, is a global priority [127,128] but at present, early warning systems for *Aedes-*borne diseases like dengue are unreliable [129], and the standard indices used to monitor *Ae. aegypti* populations are inaccurate [130].

### 5.1. Integrating Aedes Species Control with Malaria Vector Control: Uniquely African Opportunities?

Malaria cases and deaths have declined in Africa since 2000 but sustaining control toward elimination remains a major focus for African healthcare [131]. Vector control has played a central role, primarily via long-lasting insecticide treated nets (LLINs) and indoor residual spraying (IRS) [132], and this is likely to continue. What opportunities might recognition of the importance of vector control for both malaria and *Aedes*-borne arboviruses offer in Africa? Some interventions targeting African malaria vectors are unlikely to have a significant impact on *Aedes*-borne arboviral transmission because of differences in their biology. For example, LLINs primarily target nocturnal biters and zooprophylaxis targets domestic animal-biters, neither of which typically apply to *Ae. aegypti* [133,134], though the latter may be of some relevance to the more generalist feeder *Ae. albopictus* [135]. However, IRS targets indoor resting; a behaviour common not only to malaria vectors like *Anopheles gambiae* and *An. funestus*, but also to *Ae. aegypti* [133,136,137,138]. Results to date are promising [117,139,140,141], but to be effective across the target species, IRS will require careful consideration of available insecticides because in both *An. gambiae* and *Ae. aegypti*, resistance (see Section 5.2) can lead to operational IRS failure [140,142]. House screening [143] and other improvements which prevent mosquito entry [144,145] might be effective against both *Anopheles* malaria vectors and *Ae. aegypti*, although perhaps less so against the more exophilic *Ae. albopictus*.

Larval source management can be effective individually for both malaria [146] and dengue [117] but its potential as a cross-cutting intervention is limited owing to the typically different breeding sites of *Anopheles* and *Aedes*. Larval stages of *Anopheles* are usually found in ground pools or irrigated sites, especially rice paddies, while *Aedes* proliferate in manmade containers or small natural sites such as rock pools and tree holes. Nevertheless, biological and chemical larviciding formulations, such as *Bti* and pyriproxyfen, represent important tools in the arsenal for control of *Aedes* larvae [147,148] and in some settings, they may also be appropriate for *Anopheles* control [149,150], potentially offering logistical and procurement synergies.

There is currently little evidence to suggest efficacy of personal repellents for control of either dengue or malaria [117,151,152,153]. Though promising for both *Anopheles* and *Aedes* vector control [154,155], demonstration of the impact of volatile spatial insecticides on epidemiological indicators is currently lacking for malaria or arboviruses. Similarly, attractive toxic sugar baits (ATSB) might eventually prove to be effective against different diseases, although at present it is unclear whether the same bait will attract both *Aedes* and *Anopheles* species, whilst avoiding negative impacts of traps on economically-beneficial insects [156]. 

The control of *Aedes* spp. in Africa could benefit from association with already well-established national malaria control programmes. This is in line with the first pillar of the global vector control response developed by the World Health Organisation (WHO) that calls for strengthening inter- and intra-sectoral action and collaboration [157]. Following identification of disease overlaps [158] and control opportunities [61], provision of training will be a key issue to ensure that experienced malaria vector control practitioners become effective arboviral disease vector controllers. In addition, funding agreements will have to be reached to avoid diversion of resources from malaria programmes where the perceived current disease risk may be relatively low in elimination or pre-elimination settings, resources for which in many cases, are already inadequate to maintain a consistent downward malaria trajectory [137].

### 5.2. Insecticide Resistance and Challenges to Control 

Worldwide data on insecticide resistance in *Ae. aegypti* and *Ae. albopictus* are patchy, with by far the majority of data originating from Latin America and South-East Asia [19]. This paucity is readily evident in Africa, with just 18 published studies, of which three [44,64,159] were conducted over 30 years ago. The remainder involve WHO tube and larval bioassays on samples collected within the last ten years from 12 countries (Table 2). 

DDT resistance is widespread and has been reported from every country tested, and in both *Ae. aegypti* and *Ae. albopictus*. Resistance to pyrethroids (primarily permethrin and deltamethrin) appears more sporadic but there are confirmed reports in *Ae. aegypti* from mainland West, Central and East Africa. It should also be noted that *An. gambiae* doses are almost always used for assessment of pyrethroids and may underestimate resistance because *Ae. aegypti* discriminant doses are lower [160]. Pyrethroid resistance may currently be less critical than in South East Asia and Latin America [19], but in Africa appears to be emergent, and is not limited to particular regions.

Organophosphate adulticides (malathion or fenitrothion) have been less frequently tested but only in Madeira and Sudan has resistance been confirmed in *Ae. aegypti*. All studies from West Africa and islands testing carbamates (usually propoxur) have reported resistance. Studies from other areas have typically reported susceptibility, but very recent testing in Yaoundé, Cameroon, detected bendiocarb resistance in both *Ae. aegypti* and *Ae. albopictus* [71], suggesting that apparent geographical patterns may be a consequence of the limited number of studies performed to date.

Fortunately, tests of the first-line biological and chemical larvicides, *Bti* and temephos, have not detected a problematic level of resistance. In dose-response assays the highest resistance ratios (calculated from the ratio of the lethal concentration killing 50% of the test, compared to a susceptible reference strain) are from Cape Verde and suggest little resistance [161].

Susceptibility to *Bti* is expected based on its complex mode of toxicity and lack of any previous reports of resistance in *Aedes* field populations [19]. In contrast, temephos resistance is extremely common in Latin America and is found throughout Asia [19], yet apparent full susceptibility in Africa allows temephos to be regarded as a viable option for water treatment.

There is currently very limited information on the mechanisms of insecticide resistance in African populations of either key *Aedes* vector species. Knockdown resistance (*kdr*) mutations in the voltage-gated sodium channel, which can generate high levels of resistance when present in combinations [166,167], have recently been detected in West Africa, though not to date elsewhere, albeit from very limited investigations (Table 2). High frequencies of F1534C, the only *kdr* mutation known to show a worldwide distribution [19], have been found in samples from throughout Ghana [51], whilst the Latin American V1016I variant was detected in a single Ghanaian sample. In the absence of target site mutations, evidence for metabolic resistance via efficacy of synergists in bioassays or elevated activity of mixed-function oxidases, esterases and glutathione-*S*-transferases in biochemical assays has been suggested as at least a partial explanation for some resistant phenotypes (Table 2).

The source of resistance in African *Aedes* populations is less obvious than for areas of Latin America and Asia subjected to targeted control programmes. Widespread recent use of DDT and pyrethroids for IRS against malaria vectors might be one source; however, introductions, rather than simply in situ selection from local genetic variation, might be important. For example, the 1534C *kdr* mutation found in Africa is linked to a non-African, presumably immigrant, haplotype [34]. On Madeira, a multiple-insecticide resistant *Ae. aegypti* population has likely been introduced recently, possibly from Latin America [162], ready-equipped with two *kdr* mutations and overexpression of multiple pyrethroid metabolising genes [168,169].

In general, the picture of resistance suggests that viable insecticidal options remain available to target African *Aedes* populations. Yet, the potential for insecticide resistant *Ae. aegypti* and *Ae. albopictus* to be imported via human-aided movement of mosquitoes over long distances [170], rather than via slow natural migration, is a major concern, and a challenge to curbing the spread of resistance. 

## 6. Conclusions

### Major Knowledge Gaps and Recommendations

Over 60 years ago, and with considerable prescience, Mahaffy [118] argued the case for eradication of *Ae. aegypti* from Africa, writing: “A successful eradication programme of this nature carries with it results of such profound importance, not only to Africa, but also to infectible territories outside Africa, that it is impossible to over-emphasize the necessity for its initiation at the earliest possible moment”. Most would agree that the prospect for elimination of *Ae. aegypti* from Africa is not a realistic one at present, but that the need to reverse decades of neglect of arboviral disease in Africa is long overdue. 

To build an appropriate evidence base on which disease prevention and control strategies and policies can be founded, we recommend the following topics be prioritized for investigation: distribution of cases of dengue, chikungunya, Zika and other arboviruses in humans; distributions of *Ae. aegypti* and *Ae. albopictus* in Africa; updated characterization and distribution of insecticide resistance in *Ae. aegypti* and *Ae. albopictus,* with broader geographical coverage using standardized methodologies; development of context-specific *Aedes*-borne arboviral disease surveillance plans, and of outbreak prevention and vector control response strategies. 

Integral to the success of the above topics is the need for capacity strengthening in biology, diagnosis and control of arboviral disease vectors in all African nations under threat from these infections.

Additional topics or knowledge gaps that merit investigation should include the following: controlled trials of vector interventions in African settings; the pros and cons of developing *Aedes* control in liaison or integrated with malaria elimination programmes; vector competence, peridomestic behaviour and biology of African *Ae. aegypti*, especially the ecotypes, and of the other *Aedes* spp. previously incriminated or suspected as potential vectors; and occurrence and distribution of dengue, chikungunya and Zika virus infections in non-human primates and other potential ‘reservoir’ hosts.

## Figures and Tables

**Table 1:** Estimated population at risk of infection (PAR) by each arbovirus and by overlapping arboviruses in Africa, 2015. Population estimates were calculated using the methods and data sources as defined and cited in Figure 1a,b, respectively.

Infection	Estimated Population at Risk	Percentage of African Population
Chikungunya	271 million	23%
Dengue	750 million	63%
Yellow fever *	21 million	2%
Zika	406 million	34%
At least one of the above	831 million	70%

* The value for yellow fever has been adjusted to account for reductions in the population at risk following vaccination programmes.

**Table 2:** Records of insecticide resistance in *Aedes* species (1990 onwards) from African mainland countries and islands. Resistance is classified according to WHO standards as resistant (R) < 90% mortality, suspected resistance (RS) 90–97% mortality, or susceptible (S) > 97% mortality in adult (a) tube or larval (l) bioassays. Brief details of resistance mechanisms are shown where reported. Studies are ordered by species, then from North-West toward South-East. Blank cells indicate that the phenotype or mechanism was not investigated.

Study	Year	Country	Area	Species	DDT (a)	Pyr I (a)	Pyr II (a)	Carb (a)	OP (a)	Temephos (l)	*Bti* (l)	Other (l)	*kdr* Mutations	Metabolic Resistance
[162]	2013	Madeira	Funchal, Paul do Mar	*Ae. aegypti*		R	R	R	R, S				F1534C; V1016I	MFO, esterases (biochemistry); PBO, DEM significant (synergist; metabolizing genes overexpressed)
[43]	2009	Cape Verde	Santiago	*Ae. aegypti*	R	S	S	R	S					
[163]	2012	Cape Verde	Santiago, Praia	*Ae. aegypti*			R		S	S			Not detected	MFO, esterases, GSTs (biochemistry)
[63]	2009	Senegal	Dakar	*Ae. aegypti*	R	S	R, RS	R	S					
[52]	2010	Côte d’Ivoire	Abidjan	*Ae. aegypti*		S	RS, S	R, RS						
[46]	2014	Côte d’Ivoire	Abidjan	*Ae. aegypti*	R	S	RS	RS				S (DDT, Pyr)		
[164]	2012–2013	Ghana	Accra	*Ae. aegypti*	R	R	R							
[51]	2013–2014	Ghana	Widespread	*Ae. aegypti*	R	R, RS, S							F1534C; V1016I	
[51]	2013–2014	Ghana	Widespread	*Aedes aedes formosus*	R	R, RS, S							F1534C	
[37]	2011–2012	Nigeria	Lagos	*Ae. aegypti*	R	S	RS, S							
[60]	2013	Nigeria	Kwara State	*Ae. aegypti*	S	S		R						
[49]	2007	Cameroon	Widespread	*Ae. aegypti*	RS		S	S	S	S	S			
[71]	2015–2016	Cameroon	Yaoundé	*Ae. aegypti*	R	S	R	R, S	S				Not detected	Limited effect of synergist PBO
[58]	2013	Central African Rep.	Bangui	*Ae. aegypti*	R, RS		RS, S	S	S	S	S		Not detected	MFO, esterases, GSTs (biochemistry)
[49]	2007	Gabon	Libreville	*Ae. aegypti*	R		S	S	S	S	S			
[48]	2009, 2010	Sudan	Port Sudan	*Ae. aegypti*	R		RS, S	S	R, RS					
[165]	2015	Tanzania	Dar es Salaam	*Ae. aegypti*		R, RS	R, RS							
[166]	2010–2011	Mayotte	Petit Terre	*Ae. aegypti*			S			S	S	S (multiple)		
[49]	2007	Cameroon	Widespread	*Ae. albopictus*	R		RS, S	S	S	S	S			
[71]	2015–2016	Cameroon	Yaounde	*Ae. albopictus*	R	R, S	R, RS	R, RS	S				Not detected	Variable effect of synergist PBO among locales
[58]	2014	CAR	Bangui	*Ae. albopictus*	R, RS, S		R, RS, S	RS, S	RS, S	S	S		Not detected	MFO, esterases, GSTs (biochemistry)
[49]	2007	Gabon	Libreville	*Ae. albopictus*						S				
[61]	2010–2011	Mayotte	Kaweni	*Ae. albopictus*			S			S	S	S (multiple)		

Abbreviations: DDT (dichlorodiphenyltrichloroethane); Pyr (pyrethroid); Carb (carbamate); OP (organophosphates); MFO (mixed function oxidases); PBO (piperonyl butoxide); DEM (diethyl maleate); GST (glutathione S-transferase).

## Supplementary Materials

The following are available online at http://www.mdpi.com/1660-4601/15/2/220/s1, Supplementary Materials (Methods and References).
