# Supplementary material for: Aedes Mosquitoes and Aedes-Borne Arboviruses in Africa: Current and Future Threats
Source: Int J Environ Res Public Health. 2018 Jan 28;15(2):220. doi: 10.3390/ijerph15020220 (PMC5858289; doi:10.3390/ijerph15020220)
Supplement: Supplementary file 1 [file ijerph-15-00220-s001.pdf]

International Journal of
Environmental Research and Public Health

MDPI

Supplementary Materials: *Aedes* Mosquitoes and *Aedes*-Borne Arboviruses in Africa: Current and Future Threats

David Weetman [1], Basile Kamgang [2], Athanase Badolo [3], Catherine L. Moyes [4], Freya M. Shearer [4], Mamadou Coulibaly [5], João Pinto [6], Louis Lambrechts [7,8] and Philip J. McCall [1,*]

Methods

Global maps of predicted environmental suitability for chikungunya, dengue and Zika virus transmission measured on a relative scale from 0 to 1.0, for every ≈5 x 5 km, have been constructed previously [1-3]. To calculate the population at risk (PAR) from each arbovirus, we converted these maps of relative risk into binary maps of predicted infection risk presence using a threshold level of environmental suitability above which an area was considered at-risk. The threshold for dengue risk was calculated by extracting the environmental suitability values for the locations of 113 records of confirmed dengue infections [4] in Africa that have been geolocated to an area < 25 km^2. We then calculated the threshold that captured 95% of these dengue records. A 95% value was used to allow for errors in either the location and/or disease identification within the set of case reports. The same calculation was performed for chikungunya. In this case, there were not enough confirmed records from Africa alone so 394 global records, including reports from Africa, were used [5]. For Zika, there were not enough precisely-located confirmed records available globally to calculate the threshold in the same way so a threshold of 0.5 was used to identify the areas most at risk. Once the three binary maps of 'at-risk' areas were constructed, they were combined with fine scale population data for 2015 from WorldPop [6] to calculate the population living within each at-risk area.

A different approach was used for yellow fever because the extent, or risk zones, for this disease has been defined [7] and because the population at risk is radically altered in locations where the yellow fever vaccine has been implemented. A recent model incorporating yellow fever case data, vaccination coverage and national incidence figures has provided a fine scale map of predicted yellow fever incidence within these previously defined risk zones [8]. This is more informative than an environmental suitability map but does not provide values for disease risk that are comparable to the figures calculated for chikungunya, dengue and Zika. We therefore converted the incidence map to a binary map of 'at-risk' and 'no risk' by converting all incidence values greater than 0 to a value of 1. We then used vaccination coverage rates achieved by 2015 [9] and the same population data as above to calculate the number of unvaccinated individuals within each 'at-risk' area, i.e. the population at risk.

References

1. Bhatt, S.; Gething, P.W.; Brady, O.J.; Messina, J.P.; Farlow, A.W.; Moyes, C.L.; Drake, J.M.; Brownstein, J.S.; Hoen, A.G.; Sankoh, O., *et al.* The global distribution and burden of dengue. *Nature* **2013**, *496*, 504-507.
2. Nsoesie, E.O.; Kraemer, M.U.; Golding, N.; Pigott, D.M.; Brady, O.J.; Moyes, C.L.; Johansson, M.A.; Gething, P.W.; Velayudhan, R.; Khan, K., *et al.* Global distribution and environmental suitability for chikungunya virus, 1952 to 2015. *Euro surveillance : bulletin Europeen sur les maladies transmissibles = European communicable disease bulletin* **2016**, *21*.
3. Messina, J.P.; Kraemer, M.U.; Brady, O.J.; Pigott, D.M.; Shearer, F.M.; Weiss, D.J.; Golding, N.; Ruktanonchai, C.W.; Gething, P.W.; Cohn, E., *et al.* Mapping global environmental suitability for zika virus. *eLife* **2016**, *5*.

International Journal of
*Environmental Research
and Public Health*

MDPI

4. Messina, J.P.; Brady, O.J.; Pigott, D.M.; Brownstein, J.S.; Hoen, A.G.; Hay, S.I. A global compendium of human dengue virus occurrence. *Scientific data* **2014**, *1*, 140004.
5. Patching, H.M.; Hudson, L.M.; Cooke, W.; Garcia, A.J.; Hay, S.I.; Roberts, M.; Moyes, C.L. A supervised learning process to validate online disease reports for use in predictive models. *Big data* **2015**, *3*, 230-237.
6. Tatem, A.J. Worldpop, open data for spatial demography. *Scientific data* **2017**, *4*, 170004.
7. Jentes, E.S.; Poumerol, G.; Gershman, M.D.; Hill, D.R.; Lemarchand, J.; Lewis, R.F.; Staples, J.E.; Tomori, O.; Wilder-Smith, A.; Monath, T.P. The revised global yellow fever risk map and recommendations for vaccination, 2010: Consensus of the informal who working group on geographic risk for yellow fever. *The Lancet. Infectious diseases* **2011**, *11*, 622-632.
8. Shearer, F., Longbottom, J, Browne, A, Pigott, DM, Brady, OJ, Kraemer, MUG, Marinho, F, Yactayo, S, Valdelaine, EM, Aglaer, A, Fullman, N, Ray, SE, Mosser, JF, Stanaway, J, Lim, S, Reiner, RC, Moyes, CL, Hay, SI, Golding, N. Modelling existing and potential infection risk zones of yellow fever: A global analysis. *Lancet Global Health* **2017**, *In press*.
9. Shearer, F.M.; Moyes, C.L.; Pigott, D.M.; Brady, O.J.; Marinho, F.; Deshpande, A.; Longbottom, J.; Browne, A.J.; Kraemer, M.U.G.; O'Reilly, K.M., *et al.* Global yellow fever vaccination coverage from 1970 to 2016: An adjusted retrospective analysis. *The Lancet. Infectious diseases* **2017**, *17*, 1209-1217.
